# Preoperative Diagnosis of a Central Skull Base Giant Schwannoma From Tumoral Microhemorrhages Visualized on Susceptibility-Weighted Imaging

**DOI:** 10.7759/cureus.70182

**Published:** 2024-09-25

**Authors:** Adrija Krishnamoorthy, David S Bailey, Charles Specht, Brad E. Zacharia

**Affiliations:** 1 Neurosurgery, Penn State Health Milton S. Hershey Medical Center, Hershey, USA; 2 Neuropathology, Penn State College of Medicine, Hershey, USA; 3 Neurosurgery/Brain Surgery, Penn State College of Medicine, Hershey, USA

**Keywords:** clivus, giant schwannoma, intratumoral microhemorrhages, skull base, susceptibility-weighted imaging

## Abstract

A 64-year-old female presented with extensive osseous erosion of the central skull base from a large tumor, which was evaluated with a combination of CT and MRI. Susceptibility-weighted imaging (SWI) aided the correct preoperative diagnosis of giant skull base schwannoma by demonstrating intratumoral microhemorrhages, later confirmed on histology. Other imaging features on CT and MRI were not helpful to identify the schwannoma in this case.

## Introduction

Preoperative identification of skull base tumor histology using CT and MRI is an important step to help guide surgical decision-making as it applies to operative approach and establishing the goals of surgery [1]. Imaging clues such as tumor location relative to the midline of the skull base, intratumoral calcifications, and the presence of known primary tumor elsewhere can help narrow the differential diagnosis [2,3]. However, preoperative identification of a skull base tumor can be challenging in the presence of large osseous erosion, which can occur either from the bony tumor of the skull base or the tumor from one of the soft tissue structures in and around the skull base [1]. Morphologic clues such as enlargement of the neural foramen, smooth bony erosion, and dumbbell shape are some well-known features of skull base neurogenic tumors, although these features may become unrecognizable in the presence of large tumors [4,5]. Identification of a neurogenic tumor can also be complicated by its unusual origin, such as the off-petrosal branch of the facial nerve or the terminal branch of the trigeminal nerve [4,6]. In this background, a knowledge of tumor characteristics on various MRI sequences can prove useful for reaching the correct diagnosis. In this report, we describe the successful preoperative diagnosis of a giant central skull base schwannoma by identifying intratumoral microhemorrhages on susceptibility-weighted imaging (SWI) sequence, review the relevant differential diagnosis, and discuss the practical implications [7,8].

## Case presentation

**Figure 1:**
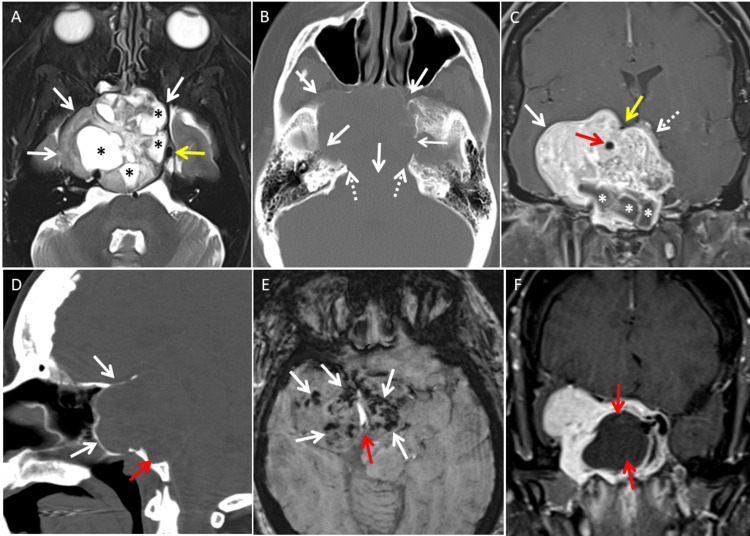
MRI and CT features of a giant skull base schwannoma (A) Axial T2-weighted TSE image shows the large central skull base tumor (solid white arrows) with multiple cystic areas (asterisk). The tumor is showing more extension towards the right middle cranial fossa. The left cavernous sinus, carotid artery, and Meckel's cave are displaced to the right (solid yellow arrow). (B) CT bone window in the axial plane shows the large osseous erosion of the central skull base (solid white arrows) with involvement of the bilateral petrous apex (dotted white arrows) and the right middle cranial fossa floor. (C) Coronal postcontrast T1 TSE with fatsat shows the large enhancing tumor occupying most of the central skull base with extension towards the right middle cranial fossa (solid white arrow). The pituitary gland is displaced to the left superolateral aspect (dotted white arrow). Cystic areas (asterisk) are observed in the inferior aspect of the solid tumor. The right cavernous carotid artery is displaced superior and apparently encircled by the tumor (solid red arrow), but a vascular groove for the posterior communicating artery in the tumor boundary is evident above the carotid artery (solid yellow arrow). (D) CT sagittal reformation of the skull shows the bony erosion of the central skull base including the major portion of the clivus (solid red arrow). The floor of the sella is absent due to the extension of the tumor into the sphenoid sinus. The thinned and displaced bony margin is evident anteriorly from the remodeling effect (solid white arrows). (E) Multiple microhemorrhages with hypointense signals are seen on the SWI scattered throughout the tumor (solid white arrows). Hyperintense signals from blood flow are evident in the right cavernous carotid artery (solid red arrow). (F) Coronal postcontrast T1 TSE with fatsat obtained after surgical resection shows the decompressed residual tumor with a large surgical cavity (solid red arrows). TSE: turbo spin echo; fatsat: fat saturation; SWI: susceptibility-weighted imaging

A 64-year-old female presented to our clinic on referral from ophthalmology after she was found to have a large intracranial lesion on MRI. MRI workup was obtained for gradual visual decline and bilateral optic atrophy discovered during routine visual screening. She had no other neurological deficits, and extra-ocular movements were full in both eyes. MRI demonstrated a large, heterogeneously enhancing tumor of the central skull base, eccentric to the right, measuring 5.3×6.7×6 cm. The tumor remained extradural with clear visualization of the dura between the tumor and the brain. Significant mass effect was exerted on the intracranial structures including the optic nerves, pituitary gland, temporal lobe, frontal lobe, brainstem, and bilateral cavernous sinuses (Figure 1). 

The tumor revealed a solid matrix with multiple cystic foci. No necrotic features were identified in the tumor. The solid matrix of the tumor demonstrated hypointense T1 and relative hyperintense T2 signals with fluid T2 signals in the cysts. Numerous microhemorrhages scattered throughout the tumor were observed on the SWI sequence. Head CT performed soon after the MRI (Figure 1) demonstrated extensive osseous erosion of the central skull base structures including the major portion of the clivus, sphenoid body, right petrous apex, right greater wing of the sphenoid, and base of the right pterygoid plate. Most of the erosive margins demonstrated smooth surface on CT. Calcification was absent in the tumor except for small areas of thin rim-like calcification near the right lateral tumor boundary and the lesser wing of the sphenoid. The right foramen ovale was unrecognizable secondary to the extensive skull base osseous erosion. A normal pituitary gland on MRI was observed near the summit of the tumor. Imaging workup for a primary malignant tumor proved unremarkable. Based on the combination of MRI and CT imaging features, differential diagnoses including chordoma, chondrosarcoma, invasive plasmacytoma, metastasis, malignant tumor of the sphenoid sinus, craniopharyngioma, and schwannoma were considered. A final imaging diagnosis of giant schwannoma was made from the presence of intratumoral microhemorrhages.

Operative notes

The patient was brought to the operating room for tumor debulking with the goal of achieving a tissue diagnosis along with decompression of the mass effect on the intracranial structures. A traditional endoscopic-endonasal transsphenoidal approach was used, with rightward expansion of the exposure via a partial medial maxillary antrostomy. The tumor proved to be firm and fibrous. After central debulking of the tumor, it was determined that the goals of surgery had been achieved and further surgical exploration could not be performed safely. Postoperative imaging demonstrated subtotal resection of the tumor with significant relief of mass effect (Figure 1). The patient made an uneventful recovery and, at three months, reported improvement in her energy level as well as cognition. However, no improvement in her vision was noticed although she denied the progression of the existing deficits.

Histology

**Figure 2:**
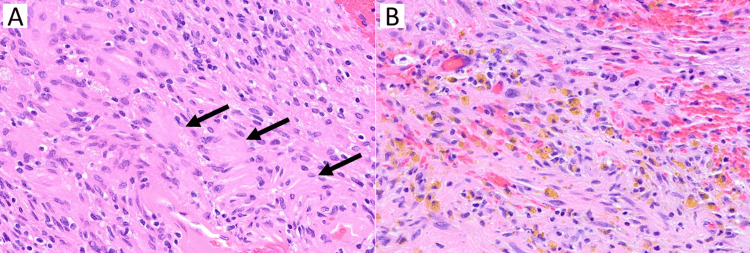
Histology of the tumor (A) H&E stain, 400×. Schwannoma with palisading Verocay bodies (black arrows). (B) H&E stain, 400×. Microhemorrhages and hemosiderin-laden macrophages (brown granules).

Histology demonstrated schwannoma, CNS WHO grade 1, without high-grade features (Figure 2). Palisading Verocay bodies were observed along with positive immunostains for S100 and SOX10. Some of the intralesional vessels showed deposits of perivascular fibrin. Further, scattered microhemorrhages and hemosiderin-laden macrophages were also observed. 

## Discussion

Extensive loss of bony structures of the central skull base can occur from tumors such as chordoma, chondrosarcoma, metastasis, plasmacytoma, schwannoma, invasive pituitary adenoma, craniopharyngioma, and extension of sinonasal tumors from the sphenoid sinus as well as nasopharynx [2-5,9,10]. The combination of MRI and CT is considered crucial for the evaluation of skull base tumors, especially the central skull base. However, challenges to an accurate preoperative diagnosis can arise from the presence of a large tumor of the central skull base as we observed in our case. The observation of intratumoral microhemorrhages on SWI sequence from MRI was the main feature that helped us to diagnose the presence of a neurogenic tumor, since previous investigators have shown the diagnostic importance of susceptibility-weighted microhemorrhages in vestibular schwannomas of the cerebellopontine angle cistern [7]. The presence of microhemorrhages has also been found to be useful to identify schwannomas in unusual locations near the skull base such as olfactory schwannoma [8]. In retrospect, the thin rim of calcification evident in the tumor in our case was thought to be displaced bone as reported previously in a neurogenic tumor [1].

Chordoma and chondrosarcoma are the two well-known tumors of the central skull base proper. Approximately one-third of chordomas occur in the skull base, and together with chondrosarcomas, they make up about 6% of skull base lesions [11]. Both tumors are well known to produce osteolytic destruction of the clivus and often mimic each other on imaging [11]. Chordomas tend to be more midline in location, have honeycomb enhancement of septations within the tumor, and demonstrate tumor calcification in approximately 30% of cases [12]. Extension of chordoma posteriorly can produce a characteristic thumbprint impression on the brainstem, considered a helpful sign for the diagnosis. In contrast, chondrosarcoma is more likely to be off-midline and has prominent T2 signals with high diffusion values in the tumor on MRI [12]. Chondrosarcomas may also demonstrate an arc and ring pattern of calcifications in the tumor [3]. The absence of these features on imaging was helpful in narrowing down the diagnosis in our case. Less commonly, pituitary adenomas are known to present as invasive tumors extending into the central skull base [2]. Visualization of a normal pituitary gland, as seen in our case, is a helpful sign to exclude invasive pituitary adenoma. Uncommonly, lymphoma or plasmacytoma may involve the central skull base. Solid and cellular tumor matrix in these tumors often results in low T2 signals without necrosis, cystic features, or hemorrhage on MRI [13]. Lytic metastatic lesions of the central skull base are an important differential consideration for lesions presenting with bony erosion or destruction. Imaging workup for a primary malignant tumor proved unremarkable. Uncommonly, craniopharyngioma can occur in the skull base in association with osseous destructive changes [14]. Until we reviewed the SWI, craniopharyngioma was considered an important diagnosis in our case in view of the presence of rim-like calcifications.

Neurogenic tumors near the central skull base may arise from cranial nerves including oculomotor, trochlear, abducens, and petrosal branches of the facial nerve. The sympathetic plexus along the internal carotid artery is another source of a neurogenic tumor near the central skull base. In the instance of giant schwannomas, extensive bony erosion can result in the destruction of critical anatomical landmarks such as neural foramen rendering neurogenic origin unrecognizable on imaging, as observed in our case [4,5,10]. Appropriate neurological deficits may point to the origin of central skull base neurogenic tumors from a specific cranial nerve [6]. The presentation of visual loss from optic nerve compression is a highly unusual presentation in a central skull base neurogenic tumor [4]. Our patient sustained visual loss due to the anterior and superior extension of the tumor towards the optic canals resulting in the compression of the optic nerves [10]. It is reported that attention to the direction of tumor extension in relationship to anatomical structures may offer a clue to the origin of neurogenic tumors near the central skull base. As an example, a neurogenic tumor arising from the greater superficial petrosal nerve near the geniculate ganglion may produce more lateralized erosive bony changes [6]. Posterior and superior displacement of the cavernous internal carotid artery from a central skull base tumor may occur from a neurogenic tumor arising from the carotid sympathetic plexus [6]. In our case, although we were able to successfully identify the neurogenic tumor on imaging, we were unable to recognize the nerve responsible for the origin of the tumor. The tumor in our case may have originated from the carotid sympathetic plexus, one of the petrosal branches of the facial nerve, or the remote terminal branches of the trigeminal nerve which are known to be distributed near the skull base including the mucosal linings of the sphenoid sinus [4]. 

## Conclusions

Knowledge of tumor imaging characteristics can help preoperatively identify central skull base tumor histology and subsequently inform treatment planning. In this case, we describe the challenging clinical and imaging presentation of a giant central skull base schwannoma correctly identified in the preoperative phase through the recognition of intratumoral microhemorrhages on the SWI sequence. 

## References

[1] Kusumi M, Oka H, Aliabadi H, Sato S, Kumabe T (2016). The appropriate surgical approach to a greater petrosal nerve schwannoma in the setting of temporal lobe edema. World Neurosurg.

[2] Battal B, Zamora C (2023). Imaging of skull base tumors. Tomography.

[3] Kunimatsu A, Kunimatsu N (2017). Skull base tumors and tumor-like lesions: a pictorial review. Pol J Radiol.

[4] Dutta G, Singh D, Singh H, Singhal G, Saran RK (2018). Atypical presentation of cystic schwannoma of the sphenoid sinus: a nonsolitary mass with osseous, intracranial and cavernous sinus invasion. Pan Afr Med J.

[5] Zięba S, Sąsiadek M, Łaczmańska I, Czapiga B, Gajdzis P, Bladowska J (2023). Giant intracerebral schwannoma of the skull base misinterpreted as a macroprolactinoma. Pol Arch Intern Med.

[6] Takase H, Araki K, Seki S, Takase K, Murata H, Kawahara N (2017). Unique diagnostic features and surgical strategy for intracranial carotid sympathetic plexus schwannoma: case report and literature review. World Neurosurg.

[7] Thamburaj K, Radhakrishnan VV, Thomas B, Nair S, Menon G (2008). Intratumoral microhemorrhages on T2*-weighted gradient-echo imaging helps differentiate vestibular schwannoma from meningioma. AJNR Am J Neuroradiol.

[8] Santhosh K, Kesavadas C, Radhakrishnan VV, Thomas B, Kapilamoorthy TR, Gupta AK (2007). Usefulness of T2*-weighted MR sequence for the diagnosis of subfrontal schwannoma. J Neuroradiol.

[9] Laigle-Donadey F, Taillibert S, Martin-Duverneuil N, Hildebrand J, Delattre JY (2005). Skull-base metastases. J Neurooncol.

[10] Bitoh S, Hasegawa H, Ohtsuki H, Obashi J, Furukawa Y, Sakurai M (1983). Schwannoma of the skull base with intracranial extension. Surg Neurol.

[11] Pamir MN, Ozduman K (2006). Analysis of radiological features relative to histopathology in 42 skull-base chordomas and chondrosarcomas. Eur J Radiol.

[12] Santegoeds RGC, Temel Y, Beckervordersandforth JC, Van Overbeeke JJ, Hoeberigs CM (2018). State-of-the-art imaging in human chordoma of the skull base. Curr Radiol Rep.

[13] Xing Z, Huang H, Xiao Z, Yang X, Lin Y, Cao D (2019). CT, conventional, and functional MRI features of skull lymphoma: a series of eight cases in a single institution. Skeletal Radiol.

[14] Lee YH, Kim SD, Lim DJ, Park JY, Chung YG, Kim YS (2009). Isolated petroclival craniopharyngioma with aggressive skull base destruction. Yonsei Med J.

